# Artificial intelligence in predicting pathogenic microorganisms’ antimicrobial resistance: challenges, progress, and prospects

**DOI:** 10.3389/fcimb.2024.1482186

**Published:** 2024-11-01

**Authors:** Yan Li, Xiaoyan Cui, Xiaoyan Yang, Guangqia Liu, Juan Zhang

**Affiliations:** ^1^ Department of Pharmacy, Jinan Fourth People’s Hospital, Jinan, China; ^2^ Pharmacy Department, Jinan Huaiyin People’s Hospital, Jinan, China; ^3^ Pharmacy Department, Pingyin County Traditional Chinese Medicine Hospital, Jinan, China; ^4^ Pharmacy Department, Jinan Licheng District Liubu Town Health Centre, Jinan, China

**Keywords:** antimicrobial resistance, artificial intelligence, machine learning, drug target prediction, pharmacology

## Abstract

The issue of antimicrobial resistance (AMR) in pathogenic microorganisms has emerged as a global public health crisis, posing a significant threat to the modern healthcare system. The advent of Artificial Intelligence (AI) and Machine Learning (ML) technologies has brought about revolutionary changes in this field. These advanced computational methods are capable of processing and analyzing large-scale biomedical data, thereby uncovering complex patterns and mechanisms behind the development of resistance. AI technologies are increasingly applied to predict the resistance of pathogens to various antibiotics based on gene content and genomic composition. This article reviews the latest advancements in AI and ML for predicting antimicrobial resistance in pathogenic microorganisms. We begin with an overview of the biological foundations of microbial resistance and its epidemiological research. Subsequently, we highlight the main AI and ML models used in resistance prediction, including but not limited to Support Vector Machines, Random Forests, and Deep Learning networks. Furthermore, we explore the major challenges in the field, such as data availability, model interpretability, and cross-species resistance prediction. Finally, we discuss new perspectives and solutions for research into microbial resistance through algorithm optimization, dataset expansion, and interdisciplinary collaboration. With the continuous advancement of AI technology, we will have the most powerful weapon in the fight against pathogenic microbial resistance in the future.

## 1 Introduction

The global pandemic of antimicrobial resistance (AMR) has claimed approximately five million lives, posing a significant health threat worldwide. Research forecasts suggest that by 2050, AMR-related mortality could rank third globally, with an estimated ten million deaths annually (de Kraker et al., 2016; Bombaywala et al., 2021; Murray et al., 2022). AMR arises when microorganisms such as bacteria, fungi, and viruses develop resistance to drugs that were previously effective in treating infections they cause. Addressing the threat of AMR and adopting preventive measures and appropriate antibiotic strategies is a crucial health care event for the future global health domain. However, identifying antibiotic resistance genes (ARGs) presents significant challenges, necessitating highly accurate treatment plans and technologies for rapid identification of current issues (Public Health Agency of Canada; in participation with the Canadian Food Inspection Agency; Canadian Institutes of Health Research; Health Canada; Agriculture and Agri-Food Canada, 2014; Hoque et al., 2020). Pathogen detection and molecular typing assays are insufficient for effective AMR monitoring. Nonetheless, high-throughput sequencing technologies offer a valuable data source for studying genetic variations in AMR.

**Figure 1:**
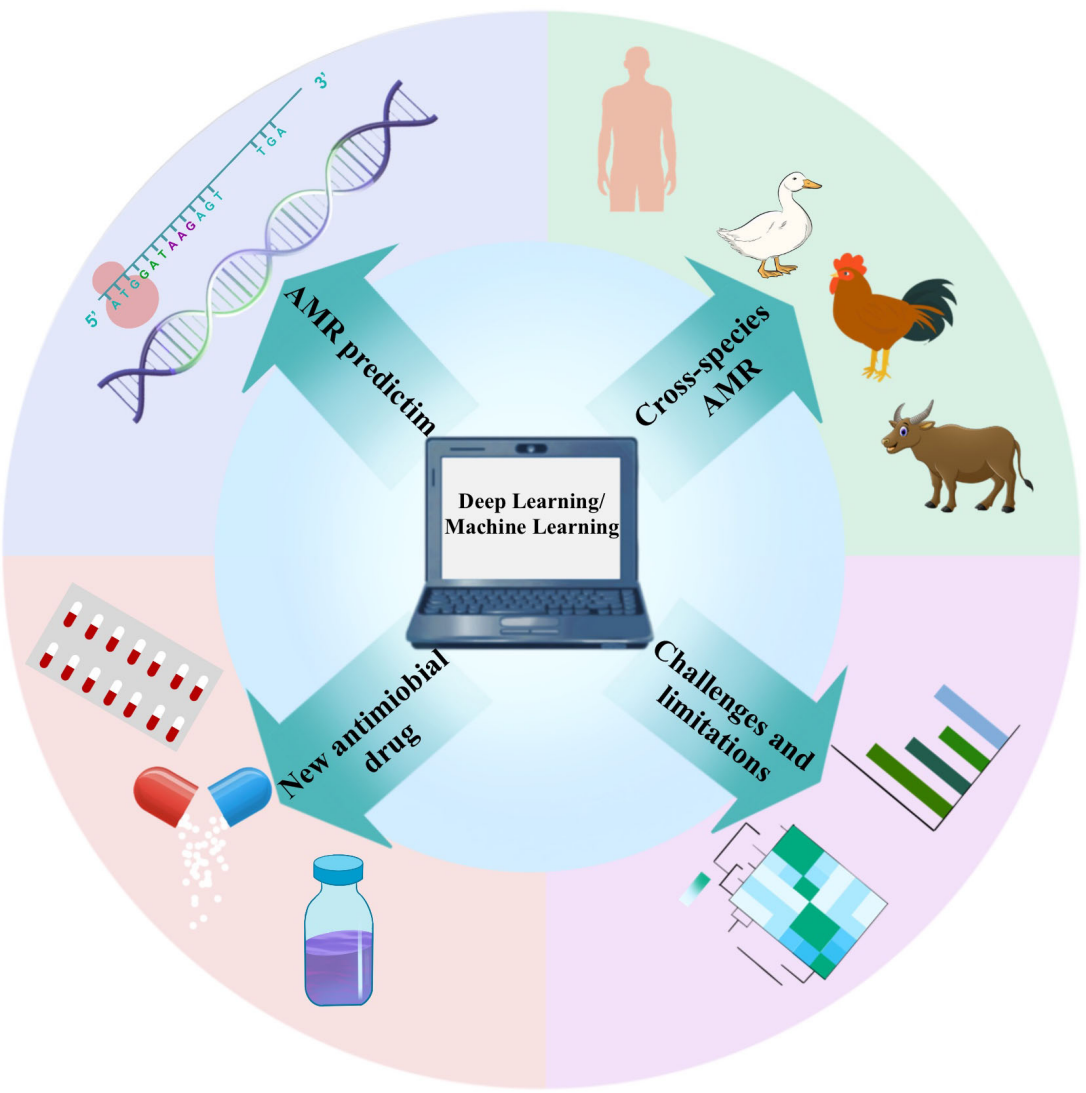
In AMR research, different application scenarios require the selection of targeted deep learning models. For AMR prediction, Convolutional Neural Networks (CNNs) and Recurrent Neural Networks (RNNs, particularly LSTM or GRU) are effective in processing genetic sequence data, extracting resistance features. The combination with CNN’s feature extraction capabilities can enhance prediction accuracy. In cross-species AMR analysis, multi-task learning models allow the sharing of parameters across different species, while fine-tuning for specific species, making them suitable for cross-species resistance analysis. For new antimicrobial drug development, Generative Adversarial Networks (GANs) and Variational Autoencoders (VAEs) are proficient at generating and optimizing drug molecules by learning molecular structure data, offering potential new antimicrobial agents. Selecting the appropriate model helps address the diverse challenges in AMR research and advances drug development and precise prediction. To address the challenges and limitations in model interpretability, using explainable AI models such as SHAP or LIME can provide explanations for the predictions of complex deep learning models, increasing transparency and trustworthiness.

The concept of Artificial Intelligence (AI) was initially proposed by John McCarthy, aimed at extending human intelligence and developing theoretical methods, technologies, and application systems (Hamet and Tremblay, 2017; Haug and Drazen, 2023). As an innovative, rapid, and cost-effective approach, AI demonstrates significant potential in addressing this challenge. It leverages computer science and vast datasets to simulate human intelligence and its problem-solving and decision-making capabilities (Paul et al., 2021). Machine Learning (ML), a crucial subfield of AI, involves designing algorithms focused on accurately predicting outcome variables. ML algorithms are trained on datasets and assessed for their predictive performance on test datasets, thereby enhancing their accuracy (Greener et al., 2022). The application of ML technology in AMR research includes sequence-based AI analysis, the design of new antibiotics, and the generation of synergistic effects in drug combinations (Anahtar et al., 2021). ML algorithms predict which microorganisms may develop resistance to specific drugs by analyzing patterns in antimicrobial drug use and resistance data, assisting healthcare professionals in making the most accurate decisions (Sakagianni et al., 2023). Furthermore, ML models play a vital role in monitoring antibiotic resistance, analyzing vast amounts of data to identify new resistance patterns and potential hotspots, aiding public health departments in more effectively addressing outbreaks of resistant infections. Deep Learning (DL), a branch of ML, represents a further deepening of AI technology in simulating complex data processing procedures. DL can more swiftly identify chemicals within large chemical libraries, aiding researchers in finding suitable antibiotic drugs (Talat and Khan, 2023). This review explores the analytical processes of ML and DL and their evolution in the era of big data. We highlight the roles of ML and DL in combating AMR, improving the development and use of antibiotics, and investigating drug-target interactions. Future AI technologies could become the most powerful weapon in the development of the antibiotic domain (**Figure 1**).

## 2 The basic process of ML in AMR

The main process of ML in AMR application should be: firstly, data collection and preprocessing. The data used mainly contain whole genome sequences (WGS) and single nucleotide polymorphisms (SNPs) with specific phenotypes (Liu et al., 2020; Ren et al., 2022a). Data preprocessing: Data preprocessing refers to the process of cleaning and formatting raw data before feature extraction, such as removing noise, handling missing values, etc. Feature extraction: Feature extraction refers to extracting useful information from the preprocessed data for model training, such as the construction of the SNP matrix. Machine learning can learn the mechanisms of AMR from DNA sequence data. There are some key features that we usually extract for subsequent analysis. These features include nucleotide k-mers, amino acid k-mers, gene content, SNP (single nucleotide polymorphism) detection, and combinations of gene content and SNP detection. Below we outline the specific steps of each feature extractor method: 1. Nucleotide k-mers: tool and parameter settings: Nucleotide k-mer counts were performed on the genome of each strain using the KMC3 tool. The minimum count of the output k-mer was set to 1 and the maximum count to 4,294,967,295. k-mer length selection: k-mer lengths of 8, 9, 10, and 11 were tested. longer k-mers were not attempted due to memory constraints. canonical conversion: Two scenarios were tested: one was to convert all non-canonical k-mers to their reverse complements to get the canonical form and compute only canonical k-mer; the other is to compute all k-mer. results show that conversion to canonical form performs better. 2. Amino acid k-mers: tool and parameter setting: use MerCat tool to count amino acid k-mers from protein FASTA sequences downloaded from PATRIC database, and set the minimum frequency of output k-mer to 1. Selection of k-mer length: count 3-mers, 4-mers and 5-mers of amino acids of each strain genome, equivalent to 9-mers, 4-mers and 5-mers. counts, equivalent to 9-mers, 12-mers, and 15-mers of nucleotides. 3. Gene content: target and method selection: predict MIC based on gene content of the strain, including all genes as features, and clustering on training data to avoid pre-training bias. Clustering and feature extraction: use MMseqs2 to cluster the amino acid sequences of all genomes in the training set, extract gene clusters and create a gene cluster feature matrix. 4. SNP: detection tools and parameter settings: use Snippy to extract SNPs by matching each genome to a reference sequence. reference sequence selection: select specific NCBI reference genomes for different species. Data Representation and Storage: One-time encoding of SNP features and storing the data in a sparse row matrix to save memory. 5. Combination of Gene Content and SNP: Feature Concatenation: Concatenation of features extracted based on the SNP detection and gene content methods described above into a vector. Data representation and storage: Due to the sparse nature of the data, the same sparse representation as for SNP detection is used. These methods identify and quantify genomic features through different bioinformatics data extraction techniques aimed at improving the prediction accuracy of microbial resistance (e.g., antimicrobial drug resistance, AMR).

Feature extraction methods for nucleotide k-mer’s can cause problems later on that are difficult to interpret due to the fact that the number of features grows exponentially as k increases. While Gene content, SNP, Combination of Gene Content and SNP, which are several feature extraction methods will be the application to a specific dataset. In order to alleviate the problems associated with k-mer length for interpretability and at the same time increase its generalizability. A viable alternative is to count amino acid k-mer in protein sequences (i.e., oligopeptide sequences). Amino acid k-mer counting utilizes the biological redundancy of nucleotide sequences to provide a more compact representation of the data. Compared to nucleotide k-mer, amino acid k-mer provides easier model interpretation and requires less computational complexity while having comparable accuracy to other feature extraction methods:(e.g., calculating nucleotide k-mer of different lengths, identifying deletions/presences of gene clusters and obtaining SNPs.) In addition, by comparing the RF, SVM, AdaBoost and XGBoost’s models to quantitatively predict AMR. Several models were compared in terms of accuracy with different feature extraction methods using XGBoost at 0.95 (other models extracted features with accuracies ranging from 0.55 to 0.80). XGBoost typically employs extreme gradient boosting regression to train the data and evaluate the model. In XGBoost, after training, each tree computes an output by comparing inputs to a series of thresholds in a hierarchical manner. Each tree attempts to correct the errors of the previous tree. The final output is the sum of the predictions of all the trees. Thus, in XGBoost, a strong learner is constructed by combining the decisions of several weak learners. And a few specific features in the XGBoost model, the amino acid k-mer has a higher precision of 0.88 (the other four are at 0.80-0.86), and the feature is stable at 0.999 (the other four are at 0.900-0.990).

We employ techniques like Chaotic Game Representation (CGR), label encoding, and one-hot encoding to transform SNPs into formats that can be understood by machine learning models. The next important steps are data preprocessing and feature extraction, including the extraction of reference alleles, variant alleles and their position information, and then the construction of the final SNP matrix. SNPs are encoded in Chaotic Game Representation (CGR), labeled encoding, and one-time encoding to be suitable for training the machine learning model. k-mer is also assigned with labels of the corresponding phenotypes and encoded (Liu et al., 2020). CGR is a graphical method for converting DNA, RNA, or protein sequences into points on a two-dimensional plane. It iteratively maps sequences in a two-dimensional space, where each nucleotide or amino acid corresponds to a specific area on the plane. This representation allows patterns and structural features within sequences to be visually presented, facilitating feature extraction and data mining through graphical analysis methods (Jeffrey, 1990). Label encoding is a method of converting categorical variables into a format understandable by models. When processing SNPs, each different allele (including reference alleles and variant alleles) is assigned a unique integer label. This approach simplifies the handling of categorical data, but in some cases, it may introduce additional ordinal information that might not be present in the original data (Nakagawa et al., 2014). Also known as one-hot encoding, this technique handles categorical data by creating a new binary column for each level of the category, where one represents presence, and zero represents absence. For SNPs analysis, this means each allele position is transformed into a vector composed of 0s and 1s, thus avoiding any assumptive ordinal relationships that label encoding might introduce (Peng et al., 2021).

In conducting ML-based AMR analysis, our choice of the aforementioned encoding techniques is based on their respective advantages and applicability. However, it’s important to emphasize that these techniques are not always the preferred choice in all situations: CGR: With its ability to intuitively display the graphical features of sequences, CGR is very useful for AMR analyses that involve complex pattern recognition (Rekadwad and Khobragade, 2017). However, CGR may be limited by computational resources in scenarios that require large-scale data processing. Label Encoding and One-time Encoding: These methods provide direct and efficient ways to handle SNPs data. Label encoding is suitable for smaller datasets and less complex models, whereas one-time encoding is better suited for complex models and large-scale datasets.

Data preprocessing, coding and feature extraction are easily implemented with different Python packages. Use of Python Packages: In our research, to implement data preprocessing, encoding, and feature extraction, we mainly rely on the following (Tennesen, 2010). Python packages: Pandas: For data processing and reading/writing CSV files, especially suitable for data cleaning, filtering, and transformation. NumPy: For efficient operations on multi-dimensional arrays, supporting a wide range of numerical computing tasks (Harris et al., 2020). Biopython: Specifically for bioinformatics, used for processing genomic sequence data, including but not limited to parsing and manipulating WGS and SNPs data. SciKit-learn: Provides a wide range of machine learning algorithms, such as SVM, random forests, and logistic regression, and also supports related functionalities for data preprocessing and feature extraction. Keras or TensorFlow: For building and training more complex deep learning models, such as convolutional neural networks (CNNs) (Shokraei Fard et al., 2022). Although our research primarily uses Python, the R language is another widely used option in the fields of bioinformatics and statistics. R offers packages such as Bioconductor for bioinformatics and genomic data analysis (similar to Biopython), Caret or randomForest for various machine learning algorithms (comparable to SciKit-learn), and rTensor and keras R as substitutes for Keras and TensorFlow for constructing and training deep learning models (Székvölgyi, 2024).

Detailed Analysis of Technical Challenges: (1). Impact of Data Scale on Running Time and Memory: Large-scale genomic data, such as WGS and SNPs, typically require long computation times. For example, datasets ranging from several GBs to TBs might need several hours to days for data preprocessing and feature extraction stages, especially on standard personal computers or workstations. Large datasets not only increase processing time but also significantly raise memory requirements. Memory needs might increase from a few GBs to tens of GBs or more when performing complex feature extraction or training deep learning models. (2). Model Complexity: Deep learning models, especially CNNs, due to their multi-layer structure, demand high graphics memory and RAM. For instance, training a CNN model with millions of parameters might require 16GB or more of graphics memory, along with corresponding CPU memory. The training time for complex models is long and requires high computational resources. Without GPU acceleration, the training process might take days to weeks, especially on standard configurations. (3). Hardware Configuration: Standard personal computers or workstations have limited capabilities for processing large datasets or training complex models. Researchers might face long wait times and frequent memory overflow issues. High-performance computing clusters and cloud computing services can significantly reduce computation time and solve memory limitations. Cloud platforms like Amazon Web Services (AWS), Google Cloud Platform (GCP), or Microsoft Azure offer scalable computing resources that can be dynamically adjusted according to demand, effectively alleviating hardware constraints. Solutions: (1). Data Dimensionality Reduction and Preprocessing: Reduce data scale and complexity before inputting data into models through dimensionality reduction techniques (such as PCA, autoencoders) and effective data preprocessing methods. (2). Model Simplification and Parameter Optimization: Reduce model complexity and lower computational resource demands by simplifying model structures or using parameter optimization techniques. (3). Parallel Computing and GPU Acceleration: Use parallel computing frameworks and GPU acceleration technologies to shorten computation times. Modern deep learning frameworks, such as TensorFlow and PyTorch, support automatic task distribution across multiple GPUs, significantly speeding up training. For large-scale data processing and complex model training, consider using high-performance computing clusters or cloud computing resources to obtain more computational and storage capacity.

In addition, various machine learning and statistical tools have been used to generate key features, e.g., Convolutional Neural Networks (CNNs) using machine learning models have been applied for feature generation for predictive AMR (Kuang et al., 2022). After data processing, a variety of machine learning models are used for predictive/classification AMR applications, such as Support Vector Machine (SVM), Logistic Regression (LR), Random Forest (RF), and DL (Arango-Argoty et al., 2018; Li et al., 2021). The core idea of these models is to construct mathematical relationships between the input features and the target labels based on the available data. Therefore, it is particularly important to select appropriate and relevant data. After several training sessions, these models are able to draw mapping relationships and learn potential nonlinear relationships. After the models are trained, they will be tested based on unseen data (i.e., test data) to validate their performance and eventually applied to real-world scenarios (Hicks et al., 2022). Models can be evaluated by a variety of metrics, including accuracy, precision, mean absolute error (MSE), etc.

With the growing problem of AMR, the screening and discovery of new drugs become especially important as there is a need to identify new compounds that can overcome known resistance mechanisms. The current state and trends of AMR also guide the direction of drug discovery research, ensuring that new treatment strategies can effectively address current and future resistance challenges. Machine learning algorithms have been used to predict pathogens’ resistance to known antibiotics, which is crucial for guiding the drug discovery process and ensuring that newly developed drugs can effectively tackle resistance issues. A deep understanding of resistance mechanisms allows drug designers to develop new molecular structures that may circumvent the resistance pathways faced by existing drugs. Here, we summarize the specific applications of different machine learning algorithms in drug screening and discovery (**Table 1**). In the field of machine learning, algorithms are extensively applied in drug screening models, encompassing a variety of types such as Support Vector Machines (SVM), K-Nearest Neighbors (KNN), Random Forest (RF), Naive Bayes (NB), CNN) and Autoencoders (AE). Support Vector Machine (SVM).In summary, these machine learning algorithms play a crucial role in antimicrobial drug screening models, not only enhancing screening efficiency but also expanding the depth and breadth of research. With continuous technological advancements, these methods are expected to yield even greater potential in the biopharmaceutical field in the future.

**Table 1:** The different machine learning algorithms and their specific applications in the context of drug screening and discovery.

Algorithm	Type	Core Strategy/Principle	Application in Drug Screening	Ref
SVM (Support Vector Machine)	Supervised Learning	Margin maximization through convex quadratic programming optimization	Predicting active compounds and molecular properties	(Huang et al., 2018; Rodríguez-Pérez and Bajorath, 2022)
KNN (K-Nearest Neighbors)	Supervised Learning	Classification based on the most similar K neighbors in feature space	Handling data scarcity and classification in early stages of drug screening	(Xie et al., 2021; Lee et al., 2022)
RF (Random Forest)	Ensemble Learning	Majority voting from multiple decision trees	Predicting drug activity based on genomic characteristics and chemical properties	(Lind and Anderson, 2019; Uddin et al., 2019)
NB (Naive Bayes)	Supervised Learning	Bayesian theorem with conditional independence assumption among features	Simplified and efficient virtual drug screening	(Chen et al., 2007; López Puga et al., 2015)
CNN (Convolutional Neural Network)	Deep Learning	Layered architecture including convolutional, pooling, and fully connected layers	Predicting toxicity of compounds and analyzing experimental drug test image data	(Hofmarcher et al., 2019; Choi et al., 2020)
AE (Autoencoder)	Unsupervised Learning	Reproducing input at output nodes through hidden layer transformation	Predicting drug-target interactions, model initialization, and feature dimensionality reduction for drug similarity evaluation	(Peng et al., 2020; Tripathi et al., 2021)

## 3 The application of ML/DL in AMR

**Figure 2:**
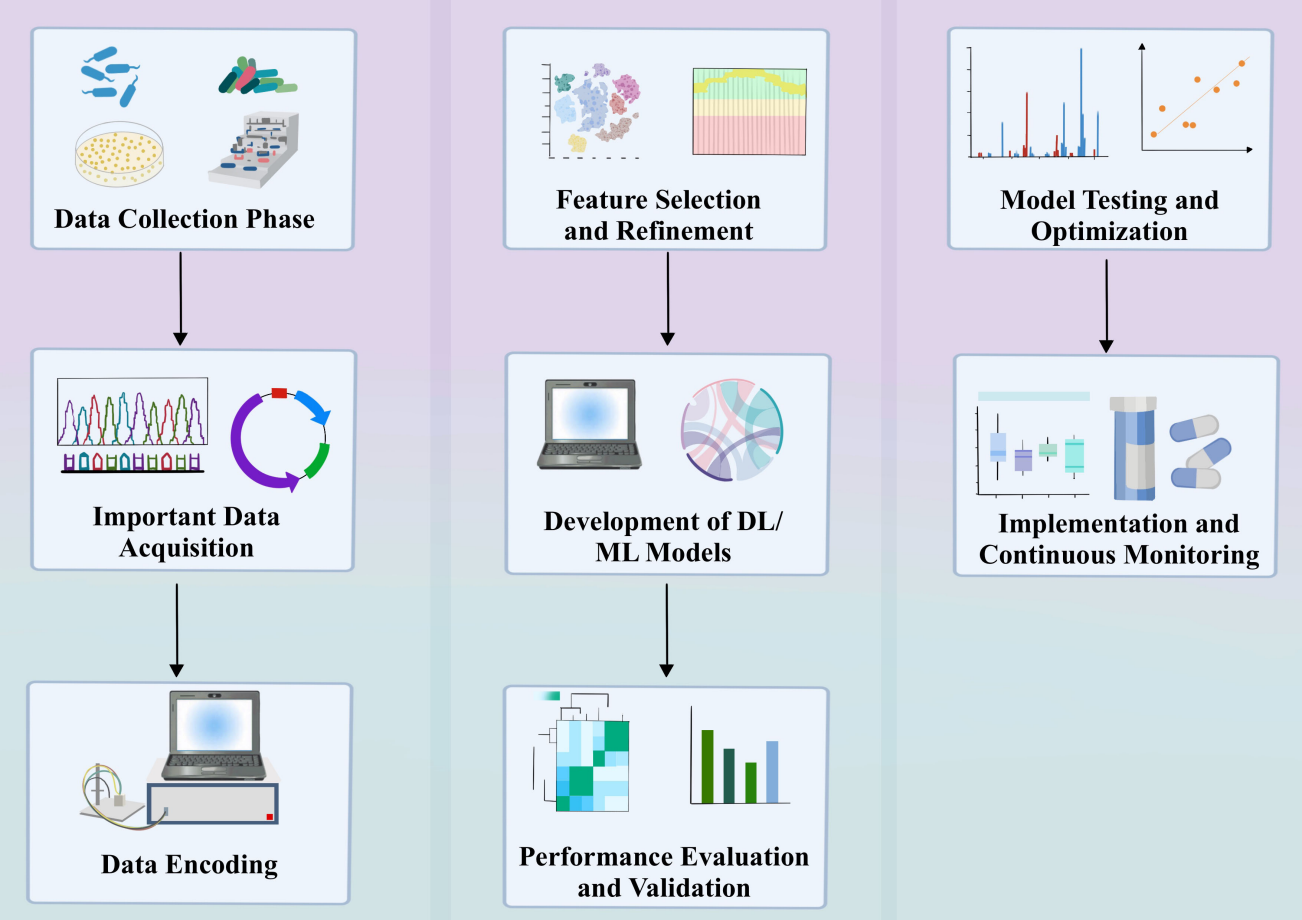
Applying DL/ML Models for AMR Identification: A Detailed Process.

ML/DL plays a crucial role in antimicrobial resistance (AMR) research, offering new methods and strategies to mitigate the impact of the AMR challenge through its powerful data processing capabilities and complex pattern recognition abilities (**Figure 2**). Deep learning technologies, particularly CNN and Recurrent Neural Networks (RNN), have shown unique advantages in learning and extracting useful information from large-scale biological datasets, which is vital for understanding and predicting the resistance characteristics of pathogenic microorganisms (Leung et al., 2014; Mongia et al., 2022).

AMR Prediction: ML/DL models are capable of processing and analyzing high-dimensional genomic data, identifying genetic markers and mutations related to antibiotic resistance (Liu et al., 2022). High-dimensional genomic data refers to datasets that contain a large number of genetic features. These datasets usually include thousands to tens of thousands of genetic markers, for instance, Single Nucleotide Polymorphisms (SNPs), which are variations in a single DNA sequence position, potentially influencing the response of microorganisms to antimicrobial agents (Uppu et al., 2018). In addition, they may include other types of genetic information, such as gene expression levels, epigenetic modifications, Copy Number Variations (CNVs), and the entire genomic variation spectrum. These data are referred to as “high-dimensional” because the number of features (dimensions) far exceeds the number of samples. The complexity of this data poses significant challenges to analysis methods, with traditional biostatistical approaches often falling short (Cai et al., 2012). In this context, machine learning and deep learning techniques become particularly important, as they can extract meaningful patterns and associations from these high-dimensional data, thereby predicting which genetic variations are related to resistance. These models predict the susceptibility of microorganisms to specific antibiotics by learning the relationships between the pathogen genome sequences and known resistance phenotypes, thereby aiding in rapid diagnosis and treatment selection (Waddington et al., 2022). Discovery of New Antimicrobial Targets: ML/DL technologies also demonstrate their potential in mining complex biological networks and metabolic pathways, revealing new targets for antimicrobial drugs. By analyzing the gene expression and protein interaction networks of pathogens, deep learning models can identify potential drug targets, providing scientific foundations for developing novel antibiotics (Bradley et al., 2015; Melo et al., 2021). Antimicrobial Peptide Design: ML/DL is further applied in the design and optimization of antimicrobial peptides (AMPs). Leveraging its ability to process sequence data, deep learning models can predict the antimicrobial activity of peptide sequences, guiding the synthesis of new AMPs with high antimicrobial efficacy and low toxicity (Cardoso et al., 2019; Wang et al., 2021). This overview highlights the significant contributions of deep learning in advancing AMR research, from predicting microbial resistance to discovering new drug targets and designing effective antimicrobial agents (Cao et al., 2023).

### 3.1 The role of ML in AMR prediction

ML has been extensively applied in the development of antimicrobial resistance research (Patel et al., 2020; Ma et al., 2022). The work of Macesic and colleagues demonstrates that ML methods can predict and identify resistant strains based on genotypic and phenotypic data, as well as improve treatment strategies and optimize clinical antimicrobial susceptibility tests for multidrug-resistant (MDR) infections (Macesic et al., 2017). Nguyen et al. utilized a vast array of non-typhoidal *Salmonella* genome data and corresponding antibiotic susceptibility spectra collected from projects such as the National Antimicrobial Resistance Monitoring System (NARMS) to develop ML methods that predict minimum inhibitory concentrations (MICs) within a ±1 2-fold dilution range, achieving an overall model accuracy of 95% (Nguyen et al., 2019). These models are designed to predict MIC values to guide response measures to outbreaks and inform antibiotic use decisions. In studies on *Mycobacterium tuberculosis*, Yang and colleagues developed models using ML techniques such as deep denoising autoencoders, analyzing data from isolates collected across 16 countries to predict their multidrug resistance (Yang et al., 2019). These studies used well-defined single nucleotide variations (SNVs) related to antimicrobial resistance to generate prediction models, demonstrating sensitivities as high as 96.3% for predicting multidrug resistance (Moradigaravand et al., 2018; Huang et al., 2023).

Moreover, ML methods have been employed to reveal microbial mechanisms of resistance to antimicrobials through the analysis of genomic data (Kim et al., 2020). These investigations focus on analyzing patient data, diagnostics, treatment, and prevention of resistance development within clinical settings. In studies of *Salmonella* whole-genome sequences (WGS), researchers developed ML models with accuracies ranging from 91% to 98% by identifying known antimicrobial resistance genes within WGS (Maguire et al., 2019). They also identified major genetic drivers of resistance in *Salmonella*, aligning with previously determined mechanisms of antimicrobial resistance. Advantages of ML over WGS Method in Predicting AMR: Improving prediction accuracy and discovering new resistance markers and hidden features: In one study, antibiotic resistance prediction in pathogens was enhanced by extending the potential resistance gene pool using pan-genome-based feature selection methods. It was found that when constructing Support Vector Machine (SVM) models by selecting relevant genes (features), the performance of genes selected using XGBoost significantly outperformed the entire genome gene set and known AMR gene set in predicting AMR (Yang and Wu, 2022). In particular, the gene set selected using the incremental method achieved an Area Under the Receiver Operating Characteristic (AUROC) curve value of over 95% in prediction performance, with the fewest number of genes required. This underscores that selecting the most appropriate gene set can significantly improve AMR prediction performance. In another study on *Mycobacterium tuberculosis (Mtb)*, genetic variations were ranked using a phylogeny-based approach, followed by training ML models to predict antibiotic resistance (Yurtseven et al., 2023). This screening is crucial and helps improve the performance of ML models. Using this approach, researchers not only identified known resistance-associated variations in a group of *Mtb* strains but also discovered new potential resistance-associated variations. This demonstrates the ability of ML methods to improve prediction accuracy and discover new resistance mechanisms (Ren et al., 2022b). Kavvas and colleagues, by integrating ML methods with genetic interaction analyses and 3D structural mutation maps, discussed the complex evolution of AMR in Mycobacterium tuberculosis against 13 antibiotics, identifying key genetic drivers of AMR. With the increase in high-quality data and the establishment of databases focused on resistance, such as the Virulence Factor Database (VFDB) (Chen et al., 2005), β-lactamase database (BLDB) (Benson et al., 2017), Antibiotic Resistance Genes Database (ARDB) (Yang et al., 2020), and the Comprehensive Antibiotic Resistance Database (CARD) (Alcock et al., 2023), these resources provide powerful tools in the fight against antibiotic resistance (Serafim et al., 2020; Jukič and Bren, 2022).

### 3.2 ML for developing new antimicrobial drugs

ML technology has shown tremendous potential in discovering AMR, fundamentally transforming the drug discovery and development process through accelerated target identification, lead discovery, preclinical, and clinical development phases (Bollenbach et al., 2009; Ferreira et al., 2020). In the field of antimicrobial drug discovery, research demonstrates how ML learns structural features of small molecules from screenings, including known antibiotics, to design novel antimicrobials (Johnson et al., 2019; Tang et al., 2022). Innovation in this area primarily focuses on developing new screening strategies that enhance sensitivity through genetically engineered microbial strains and applying novel chemical structure representations to improve the learning of chemical property algorithms with ML (Stokes et al., 2020). Johnson et al. devised a screening strategy aimed at discovering biochemical inhibitors for essential genes in *Mycobacterium tuberculosis* by first creating a genetic library composed of knockdowns of these essential genes, followed by screening 50,000 compounds against these subtypes (Johnson et al., 2019; Osman et al., 2022). Using known anti-tuberculosis antibiotics as a reference, they employed supervised ML classification analysis to identify novel chemical inhibitors for existing drug targets (such as DNA gyrase, mycolic acid synthesis, and folate metabolism) and new drug targets (such as the EfpA efflux pump), which were validated in wild-type cells. Stokes et al. conducted biochemical screenings on approximately 2,300 chemically diverse compounds to detect antimicrobial activity against *Escherichia coli*, and used the resulting data to train a deep learning ML model to predict antimicrobial activity solely from chemical structure. These authors applied the ML model to a drug repurposing hub, discovering SU3327 (a c-Jun N-terminal kinase inhibitor) and validating it as an effective inhibitor against ESKAPE pathogens and multidrug-resistant organisms (Stokes et al., 2020). Applying this ML model to more than 107 million molecules in the ZINC15 database, they identified eight putative antimicrobial compounds with structures distinct from known antimicrobials, demonstrating the potential of ML in propelling the discovery of lead compounds. Additionally, ML methods have been utilized to assist in the design and optimization of antimicrobial peptides (AMPs), proving effective against resistant pathogens (Wu et al., 2014). AMPs, being natural substrates for ML algorithms due to their complete representation by peptide sequences, are typically short (<30 amino acids), making them amenable to oligopeptide synthesis and enabling a comprehensive screening of chemical structure space (Porto et al., 2018). Wu et al. designed DP7, a novel 12-amino acid AMP with activity against *Staphylococcus aureus*, by training an ML model with multiple 12-amino acid AMPs to estimate the contribution of amino acids at each position to overall antimicrobial activity (Wu et al., 2014; Shi et al., 2019). They synthesized this AMP and demonstrated its *in vivo* efficacy against both drug-sensitive and resistant *Staphylococcus aureus*. Similarly, Porto et al. applied an ML genetic algorithm to peptides derived from the pomegranate plant to design optimized plant template AMPs with antimicrobial activity. This analysis led to the discovery of a new AMP, Avian Defensin 2, proving its *in vivo* efficacy against various pathogens (Lluka and Stokes, 2023). As ML methods continue to evolve and improve, the future of antimicrobial drug development is poised for a significant leap forward, becoming a crucial tool in combating AMR (Porto et al., 2018; Zhang et al., 2019).

## 4 Application of artificial intelligence in cross-species AMR

In recent years, the application of artificial intelligence (AI) technology in the medical field has been increasing, especially showing great potential in the prediction of cross-species antimicrobial resistance (AMR). This article synthesizes multiple studies to explore the application achievements and challenges of AI in AMR prediction. (1) Challenges and Methods in Cross-Species AMR Prediction: Application of Whole Genome Sequencing (WGS) in *Acinetobacter baumannii* Resistance Prediction: A study analyzed β-lactamase resistance genes and non-β-lactamase resistance genes in isolates of *A. baumannii* from human and non-human sources using WGS data and the ResFinder database. The study identified genes associated with resistance to various antibiotics (including aminoglycosides, tetracyclines, etc.), demonstrating the effectiveness of WGS in predicting cross-species AMR (Wareth et al., 2021). (2) Predicting E. coli AMR in Livestock and Companion Animals: A study used machine learning models to enhance AMR monitoring of pathogens such as *Escherichia coli*, utilizing sequencing information to accurately predict resistance phenotypes, thereby avoiding the need for antibiotic sensitivity testing and potentially identifying new AMR gene determinants in the process. This study also emphasized the complexity of genotype-phenotype relationships and their variations with different antibiotics, host animals, and other factors (Chung et al., 2023). (3) Rapid Antibiotic Resistance Sequence Prediction Based on Large-Scale MALDI-TOF Data: When exploring multidrug resistance phenomena in *Staphylococcus aureus*, a prediction model constructed using XGBoost-based multi-label learning based on MALDI-TOF mass spectrometry data enabled rapid prediction of dual resistance. This study demonstrated the potential of AI in accurately predicting antibiotic sensitivity of specific pathogens, which is crucial for rational antibiotic use and treatment optimization (Zhang et al., 2022).

Despite the significant potential of AI technology in cross-species AMR prediction, it still faces many challenges: Data Quality and Availability: High-quality, large-scale datasets are the foundation for training effective AI models. However, relevant AMR data may be extremely limited for rare microbes or isolates from atypical environments. Complexity of Genotype-Phenotype Relationships: The relationship between AMR genotypes and phenotypes is very complex, especially when considering the effects of different antibiotics, host animals, and other environmental factors.AI models need to handle this complexity to improve prediction accuracy and reliability. Variability in Cross-Species Biological Environments: Differences in microbial environments between different species may lead to variations in gene expression and resistance mechanisms, posing additional challenges for the design and optimization of AI models.

In conclusion, AI shows significant potential in cross-species AMR prediction, but it also faces challenges such as data quality, complexity of genotype-phenotype relationships, and variability in cross-species biological environments. These studies not only confirm the effectiveness of AI technology in predicting and monitoring AMR but also emphasize the need for future research to further explore AMR mechanisms and optimize prediction models across different species, highlighting the importance of interdisciplinary cooperation.

In the field of AMR, the application of ML technology is gradually showing great potential and benefits. First of all, while traditional antibiotic susceptibility testing is usually time-consuming and requires specialized bioinformaticians for data processing, the introduction of AI and ML technologies can dramatically reduce diagnostic time and improve accuracy. For example, the combination of flow cytometry and ML modeling has successfully reduced the diagnostic time for antimicrobial susceptibility testing to 3 hours, which greatly improves the speed and accuracy of clinical decision-making. Second, AI and ML also play an important role in genomic data management. By optimizing the genomic data processing process through AI technology, antibiotic resistance genes and their mutations can be identified more quickly, providing a more accurate basis for customized treatment strategies. And in clinical practice, some studies have demonstrated the effectiveness of AI-based antimicrobial drug treatment strategies, such as the use of AI to predict the optimal antibiotic use regimen in sepsis treatment, in order to improve treatment efficacy and reduce patient hospitalization time. In addition, the application of AI and ML technologies can improve the accuracy and efficiency of traditional phenotype detection methods. By training ML models, the accuracy of traditional phenotypic detection methods such as the Phoenix system has been significantly improved, making it a more reliable and efficient diagnostic tool. The application of AI and ML technologies has also played an important role in the discovery and production of new antibiotics. Through computer simulation and deep learning algorithms, AI can predict the structure and efficacy of new antibiotic molecules, accelerating the process of new drug development. In fact, about 14 new antibiotics have been successfully developed and approved since 2014, with the application of AI technology making a significant contribution to this progress. Finally, in monitoring and predicting AMR trends, AI and ML models can identify new resistance trends and transmission patterns by analyzing genome-wide sequence data. By monitoring known disease-causing resistance genes, AI can rapidly identify emerging resistance variants and thus take timely and appropriate countermeasures. A small number of comprehensive ML studies combined with experimental validation have demonstrated their effectiveness in confirming the accuracy of ML predictions, as well as the ability to discover previously unknown determinants of AMR or substrate activity. Initial ML models are further optimized by validating gene expression and experimentally studied gene sequences, improving predictive performance and increasing interpretability. This depth of understanding is critical to support the development of ML models based on known mechanisms and to help minimize the impact of genomic variants that do not contribute to, but are associated with, resistance phenotypes.

In summary, AI and ML technologies play an important role in antimicrobial resistance management, but also face many challenges such as data quality, model complexity, and interdisciplinary collaboration. Future studies should further explore AMR mechanisms in depth, optimize prediction models, and enhance interdisciplinary collaboration to maximize the benefits of these technologies in clinical practice.

## 5 The limitations of ML applications in AMR

While AI technologies have shown promise in unraveling AMR, facilitating rapid diagnostics, and more accurate treatments, they are accompanied by significant challenges (Sunuwar and Azad, 2021; Rabaan et al., 2022; Yurtseven et al., 2023). The mechanisms of antibiotics and drugs are not fully understood, particularly in the case of emerging diseases. Furthermore, understanding these mechanisms becomes increasingly difficult with mutations and other changes. Resistance behavior varies from the cellular level to the microbial community level (Lau et al., 2021; Májek et al., 2021; Wang et al., 2022). For instance, bacterial subpopulations may persist in response to certain cellular stressors or antibiotics, and during biofilm formation, the genomic capacity for resistance can be rapidly enhanced through horizontal gene transfer (HGT) (Olsen, 2015; Kherabi et al., 2024). Even with complete genome sequences, these types of challenges are difficult to address. Consequently, AI models may struggle to learn the underlying mechanisms of resistance evolution.

Currently, most AI models handle individual genes or gene sequences, i.e., univariate analysis. While these models are accurate in prediction, some phenotypes may result from combinations of genes or features, producing nonlinear composite effects (Tunstall et al., 2020; Bombaywala et al., 2021). For example, the combination of metal and antibiotic resistance genes may lead to specific antimicrobial drug resistances. The maintenance and spread of AMR are considered to be amplified through associations. Although metal resistance may not directly affect antibiotic resistance, its combination with antimicrobial drug resistance genes has shown an enhanced response to antimicrobial drug resistance. Since most current AI models use single, independent features, capturing these types of synergistic or associative effects is challenging (Li et al., 2017). Research on the combined effects of features or genes is scarce, and designing models capable of analyzing multivariate feature interactions faces significant hurdles. Furthermore, the classification of antimicrobial drug resistance has traditionally been a binary categorization of susceptibility or resistance (Aytan-Aktug et al., 2020). Although ML/DL models have shown good accuracy in diagnosing highly resistant or susceptible genes, including an intermediate category could lower their precision (Li et al., 2018). Incorporating an intermediate phenotype category into model design could make outcomes more effective in practical applications but also encounters challenges, such as the lack of clear standardized boundaries between susceptible, intermediate, and resistant cases, and the evolving definitions of susceptibility and resistance (Dutt et al., 2022). The scarcity of intermediate isolates may lead to imbalanced training and testing datasets, thus affecting the accuracy of model assumptions or outcomes (Wang et al., 2022). Data availability and quality are major challenges in AMR research. Data on AMR, especially for less common microbes or those isolated from unusual environments, is limited, making it difficult to train effective machine learning models. Furthermore, the quality of data used to train machine learning models can significantly impact performance. Low-quality data (e.g., noisy or contaminated data) may lead to inaccurate results (Cusack et al., 2019; Ren et al., 2022b). Overfitting of models, especially when they are highly complex, is another critical challenge, meaning they may perform well on training data but poorly on unseen data. Careful tuning of model complexity to achieve good generalization performance is crucial.

The scarcity of data in AMR research, particularly the lack of data on rare microorganisms or strains in atypical environments, significantly limits the generalization ability of models. In the study of specific pathogens or rare microorganisms, there is often insufficient sample data, making it difficult to train models with good generalization capabilities. To address this issue, previous studies have proposed the use of data augmentation techniques or the generation of synthetic data using Generative Adversarial Networks (GANs). These methods can, to some extent, alleviate the challenge of insufficient data. In addition to data scarcity, data quality issues (such as noise or contamination) can also affect the accuracy of models. AMR datasets are often poorly standardized, and measurement errors or data contamination can lead to overfitting or bias in models. We will explore how data preprocessing, outlier detection, and regularization methods can address these issues, while further emphasizing the importance of data cleaning and standardization in improving model performance. In AMR research, data imbalance is particularly evident, especially in the classification of intermediate phenotypes. For example, there are more samples of highly resistant or highly sensitive strains, while “intermediate” samples are less common. This imbalance may cause models to favor the dominant categories during training, neglecting minority class samples. We will discuss how to address data imbalance issues through techniques such as under sampling, oversampling, and SMOTE, as well as class weight adjustment strategies. The complexity of biological systems also presents significant challenges for AMR prediction. The mechanisms of antibiotic action and bacterial resistance are not fully understood, especially in the context of emerging diseases and complex resistance conditions. As phenomena such as bacterial mutations, horizontal gene transfer (HGT), and biofilm formation occur, models must handle more complex environments. The formation of biofilms can accelerate the spread of resistance genes, making it difficult for existing models based on single genes or features to accurately predict the evolution of resistance under multi-gene, multi-environment conditions. Most AI models currently use univariate analysis (e.g., single genes or gene sequences); however, resistance may arise from the combined effects of multiple genes or features. This non-linear composite effect poses significant challenges for model design. For instance, the combination of metal resistance genes and antibiotic resistance genes may lead to specific resistance, while metal resistance genes alone may not directly affect resistance. Additionally, bacterial survival strategies and resistance evolution under specific pressures may cause the same bacteria to exhibit different resistance responses in different environments, making it difficult for models to predict global resistance patterns. Current AI models are insufficient in addressing these dynamic changes at the microbial community level. Aside from the complexity of biological systems, the design limitations of existing ML/DL models also affect their application in AMR. Most resistance prediction models adopt binary classification (i.e., sensitive or resistant); however, in real-world applications, the presence of “intermediate” phenotypes complicates classification. We will explore the practical value of intermediate classification in clinical applications, especially in predicting and guiding antibiotic treatment. However, due to a lack of standardized classification boundaries and scarce data samples, existing AI models may experience performance degradation and data imbalance issues when incorporating intermediate classification. How to improve model architectures to accommodate ternary classification or continuous phenotype prediction will be a direction worth studying in the future. The complexity of biological systems and the high dimensionality of data often lead to increasingly complex AI model designs, which in turn increase the risk of overfitting. Overly complex models may perform well on training data but perform poorly on unseen data. How to prevent overfitting by adjusting model complexity, using regularization techniques, and employing cross-validation will be an important research direction.

In summary, despite the significant potential of AI in combating AMR, overcoming these challenges to maximize the application’s benefits requires further research and interdisciplinary collaboration.

## 6 Conclusion

The application of ML and deep DL in predicting AMR in disease-causing microorganisms not only represents a major advancement in the field of bioinformatics, but also has important implications for drug development. These advanced techniques provide new avenues for identifying novel resistance mechanisms and accurately predicting microbial susceptibility to antibiotics, providing a basis for the discovery of potential antimicrobial drug targets. ML and DL approaches have demonstrated their unique strengths in processing sequence data, learning complex patterns, and analyzing specific genes and mutations, providing a tool for identifying new resistance-associated biomarkers. The application of these technologies has greatly advanced the understanding of AMR mechanisms and provided a scientific basis for designing new antibiotics and therapeutic strategies. With the increase in computational power and optimization of algorithms, it is expected that the application of ML and DL in the field of AMR prediction will be further expanded, while the accuracy and reliability of models will be improved through the integration of multiple types of data. In addition, the development of interpretable models will help scientists better understand the biological mechanisms of AMR, thereby facilitating the discovery and development of new antibiotics. Through interdisciplinary collaborations, algorithmic innovations, and dataset expansion, more progress is expected to be made in predicting antimicrobial drug resistance in pathogenic microorganisms.

CNN has demonstrated exceptional performance in the field of image processing, and in recent years, its architecture has been applied to the analysis of microbial genome data for predicting antimicrobial resistance (AMR). Specifically, CNN can automatically extract features from complex, high-dimensional genome data, significantly reducing the need for manual feature engineering. Through layer-by-layer convolution and pooling, CNN can identify and capture local patterns in microbial genomes, such as specific antimicrobial resistance gene fragments or sequence motifs. In the task of automatic classification of resistance genes, CNN, trained on large-scale genome sequence data, can accurately predict bacterial resistance to different antibiotics. Studies have shown that deep CNN architectures can capture long-range dependencies within gene sequences, dependencies that are difficult to detect using traditional sequence analysis methods. Additionally, CNN can be combined with other feature extraction algorithms, such as Recurrent Neural Networks (RNN), to further enhance the identification of resistance genes.

Generative Adversarial Networks (GAN) have recently demonstrated powerful capabilities in generating high-quality synthetic data, particularly for data-scarce fields. In AMR research, GAN’s primary application lies in generating scarce microbial genome data, thereby providing more training data for deep learning models. This is crucial for antimicrobial resistance prediction, as resistance data for many microbial samples, especially for rare or emerging pathogens, is relatively scarce. Through adversarial training, GAN models generate new antimicrobial resistance genome sequences, simulating real-world resistance gene variations. This method can enhance existing datasets, mitigating overfitting issues caused by the limited availability of samples. Specifically, GAN can generate synthetic resistance gene sequences within AMR data, enabling models to be trained across a broader range of data, thereby improving their generalization capabilities. Some studies have successfully used GAN to generate bacterial genomes with resistance mutation characteristics, and these synthetic datasets have significantly improved the accuracy of subsequent models in predicting resistance evolution trends. Variational Autoencoders (VAE) are powerful generative models capable of learning latent low-dimensional representations from high-dimensional data, making them well-suited for handling complex genome data and understanding resistance mechanisms. In AMR research, VAE can utilize dimensionality reduction techniques to identify complex relationships between gene networks, thereby enhancing the prediction of resistance genes. By learning the latent distribution of genome data, VAE can extract latent features of resistance genes and effectively capture associations between different genes. This approach addresses the issue of gene interaction effects that are often overlooked in univariate analysis models. For example, VAE can recognize nonlinear combinatorial effects between resistance genes, which are often challenging to model in traditional methods. Recent studies have shown that combining VAE with a multi-task learning framework allows simultaneous prediction of multiple resistance gene features, enabling the model to perform well in addressing multi-gene resistance challenges.
